# miR-377-dependent BCL-xL regulation drives chemotherapeutic resistance in B-cell lymphoid malignancies

**DOI:** 10.1186/s12943-015-0460-8

**Published:** 2015-11-04

**Authors:** Sayer Al-harbi, Gaurav S. Choudhary, Jey Sabith Ebron, Brian T. Hill, Nagarajavel Vivekanathan, Angela H. Ting, Tomas Radivoyevitch, Mitchell R. Smith, Girish C. Shukla, Alex Almasan

**Affiliations:** Departments of Cancer Biology, Cleveland, OH 44195 USA; Genomic Medicine Institute, Cleveland, OH 44195 USA; Quantitative Health Sciences, Lerner Research Institute, Cleveland, OH 44195 USA; Department of Hematology and Oncology, Cleveland Clinic, Taussig Cancer Institute, Cleveland, OH 44195 USA; Department of Human Cancer Genomic Research, King Faisal Specialist Hospital and Research Cancer, Riyadh, 11211 Saudi Arabia; Department of Pathology, Case Western Reserve University School of Medicine, Cleveland, OH 44106 USA; Department of Biological, Geological, and Environmental Sciences, Center for Gene Regulation in Health and Disease, Cleveland State University, Cleveland, OH 44115 USA

**Keywords:** BCL-xL, BCL-2 family gene expression, Chronic lymphocytic leukemia, micro-RNA, miR-377, 14q32 miRNA cluster

## Abstract

**Background:**

BCL-xL is an anti-apoptotic BCL-2 family protein that inhibits apoptosis and is overexpressed in many cancers. We have reported that acquired resistance to the BCL-2 inhibitor ABT-199 (venetoclax) is associated with increased BCL-xL expression. Yet, how BCL-xL mediates chemoresistance in hematopoietic malignancies is not clear. This finding may help in design of new strategies for therapeutic intervention to overcome acquired chemoresistance mediated by BCL-xL.

**Results:**

We now show that the increased BCL-xL expression was inversely correlated with that of miR-377 in ABT-199-resistant cells. This finding was also extended to a panel of B-cell lymphoid lines and primary chronic lymphocytic leukemia (CLL) cells. miR-377 suppressed BCL-xL expression by recognizing two binding sites in the BCL-xL 3’-UTR. Mutation of these two miR-377 consensus-binding sites completely abolished its regulatory effect. Expression of a miR-377 mimic downregulated BCL-xL protein expression and significantly increased apoptotic cell death. Expression of a miR-377 inhibitor restored BCL-xL protein expression and limited cell death caused by the hypomethylating agent 5-azacytidine. Thus, miR-377-dependent BCL-xL regulation drives acquired therapeutic resistance to ABT-199. We further show that CLL patients who received a diverse array of chemotherapy regimens also had significantly higher BCL-xL and lower miR377 expression, indicating that exposure to chemotherapy might trigger transcriptional silencing of miR-377, which results in high levels of BCL-xL. Importantly, CLL patients with high BCL-xL/low miR-377 expression had an advanced tumor stage. Moreover, the high BCL-xL expression correlated with short treatment-free survival in 76 CLL patients. miR-377 is located at 14q32 in the DLK1-DIO3 region, which encodes the largest tumor suppressor miRNA cluster in humans. Examination of five additional 14q32 miRNAs revealed that the majority were significantly down-regulated in most CLL patients as well as in ABT-199-resistant cell lines. Remarkably, four of these miRNAs had significantly decreased expression in chemotherapy-treated CLL patients as compared to those untreated. These findings indicate a reduced expression of multiple miRNAs that may reflect a global silencing of this miRNA cluster in therapy-resistant lymphoid cells.

**Conclusions:**

These findings reveal a novel mechanism by which down-regulation of miR-377 increases BCL-xL expression, promoting chemotherapy resistance in B-cell lymphoid malignancies.

**Electronic supplementary material:**

The online version of this article (doi:10.1186/s12943-015-0460-8) contains supplementary material, which is available to authorized users.

## Background

The BCL-2 family proteins regulate apoptosis primarily on the mitochondrial outer membrane through the intrinsic apoptotic pathway [1, 2]. These proteins are divided into three classes based on their BCL-2 homology (BH) domains (BH1-BH4) and function [3]: anti-apoptotic [BCL-2, BCL-xL, BCL-W, MCL-1, BCL2A1 (BFL-1, A1), and BCL-B], pro-apoptotic multi-domain effectors (BAX and BAK), and BH3-only proteins (e.g. BIM, PUMA, and NOXA). Inhibition of apoptosis is accomplished by sequestering pro-apoptotic proteins and thus preventing mitochondrial outer membrane permeabilization [4].

Blocked apoptosis is a hallmark of treatment-resistant cancers and thus it suggests that BCL-2 family members have potential as clinical biomarkers [1, 5]. In fact, several studies have linked BCL-2 family expression and response to chemotherapy in different types of cancers. It has been reported that patients with cancers highly “primed” to cross the apoptotic threshold exhibit superior clinical responses to chemotherapy [6]. For chronic lymphocytic leukemia (CLL), high BCL-2 and MCL-1 expression levels have been reported to mediate resistance to chlorambucil, fludarabine, and rituximab [7–9]. Although numerous studies have focused on the role of BCL-2 or MCL-1 in CLL, the role of other anti-apoptotic proteins and their contribution to clinical outcome is not clearly defined. Most importantly, pharmacologic inhibitors of BCL-2 family proteins are poised for widespread clinical use, so there is an immediate need for development of markers that can rationally direct and better personalize the use of these agents in the clinic [10]. We have developed an anti-apoptotic BCL-2 family expression index that can predict the response of hematological cells, including CLL, as well as solid tumor malignancies, to the rationally designed BCL-2 family inhibitor, ABT-737/ABT-263 (navitoclax) [11]. ABT-199 (venetoclax), a second-generation, rationally designed inhibitor that was re-engineered to bind selectively to BCL-2, shows anti-tumor activity in primary tumor cells and xenograft models [12, 13]. Phase I clinical trials with ABT-199 have had high patient response rates that include many complete responses [14].

Lymphoid malignancies, most commonly derived from B-cell precursors include more than 40 distinct tumor types, varying widely in phenotype and clinical behavior [15]. CLL, the most common leukemia in the Western world [16], is characterized by an expansion of small mature B cells in blood, lymph nodes, and bone marrow. Its heterogeneous clinical course [17, 18] has led to a search for markers that can predict disease progression to allow better management of the disease. Mutational status of the immunoglobulin heavy chain variable (IGHV) region dichotomizes CLL patients into two risk categories: those with unmutated IGHV have an unfavorable prognosis, whereas patients with mutated IGHV tend to have a more favorable prognosis. ZAP70 and CD38 expression can serve as a surrogate for an unmutated IGHV gene, thus functioning as prognostic markers [19, 20]. Despite of their clinical value, there are technical difficulties that preclude optimal use of these markers, such as standardization and reproducibility [21, 22]. In addition, p53 deletion is a well-established marker of shorter survival and chemotherapy resistance [23]; however, it is present in only a small percentage of patients with CLL at the initial diagnosis [24, 25]. Overall, existing established prognostic markers fail to predict clinical outcome in a considerable number of patients with CLL [24]. Moreover, it is difficult to integrate the results of these various markers to assess the overall risk in an individual patient [22]. Thus, developing additional markers for CLL is of considerable interest as they may indicate inherent biologic differences that may be amenable to targeted therapeutic intervention.

MicroRNAs (miRs) are small non-coding regulatory RNAs that bind to a specific target mRNA through a sequence that is complementary primarily to the 3’-UTR of the target mRNA. They have roles in many underlying cancer processes, including proliferation, apoptosis, and invasion [26, 27]. miRNAs are very stable and are found in body fluids such as plasma, serum, and urine, therefore cancer-specific miRNAs could potentially be used as a tumor molecular signature to track and predict cancer progression and to guide treatment [28].

Here, we report that high BCL-xL expression inversely correlated with decreased levels of a newly identified miR-377. Mutational and functional analyses validated BCL-xL as a direct target of miR-377. Moreover, we show that BCL-xL/miR-377 regulation in diffuse large B-cell lymphoma (DLBCL) cells drives acquired therapeutic resistance to ABT-199 and is associated with advanced tumor stage in CLL patients. Collectively, these data support a model in which co-regulation of BCL-xL and miR-377 mediates a novel mechanism of acquired therapeutic resistance in B-cell lymphoid malignancies.

## Results

### High BCL-xL expression mediates chemotherapy resistance to ABT-199 in B-cell lymphoid malignancies

**Fig. 1:**
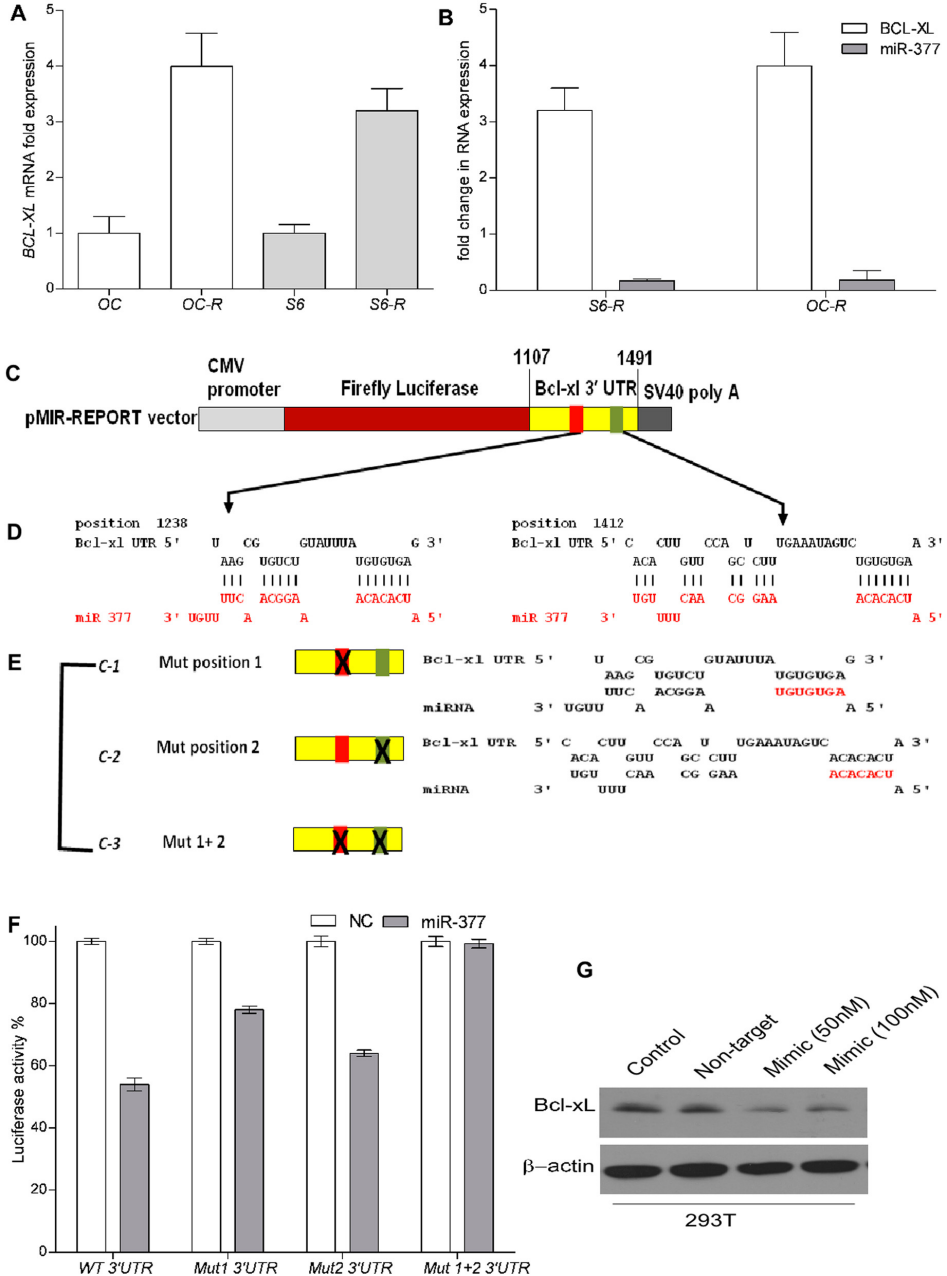
BCL-xL is a direct target of miR-377. **a** BCL-XL expression, as determined by quantitative RT-PCR in ABT-199-resistant (ABT-199R) OC-R and S-6R relative to those in parental OCI-LY19 (OC) and SU-DHL-6 (S6) and cell lines. **b** RT-PCR analysis of *BCL-xL* and miR-377 expression levels in: parental OC, S6, and ABT-199R derivative OC-R and S6-R cells. **c** Schematic representation of firefly luciferase reporter constructs containing the 384-nucleotide sequence from the *BCL-xL* 3’-UTR and the corresponding binding regions for miR-377. **d** miR-377 target sequence base pairing in *BCL-xL* 3’-UTR. **e** Schematic of the mutant constructs. **f** Luciferase reporter activity in CHO-K1 cells co-transfected with *BCL-xL* WT-3’-UTR or MUT-3’-UTR constructs: mutants C1, C2, and C3 (double-mutant) and miR-377 or negative control (NC) mimics (10 nM) as indicated. **g** BCL-xL levels were assessed by immunoblot in 293 T cells transfected with miR-377 mimic at two concentrations (50 and 100 nM). β-actin was used as a loading control

ABT-199 is a rationally designed selective BCL-2 inhibitor that has shown promising results in clinical trials, so understanding the mechanism of acquired resistance to it is important. To this end, we generated ABT-199-resistant (ABT-199R) cell lines from initially sensitive SU-DHL-6 and OCl-LY19 DLBCL cells derived from germinal center B cells (GCB). These GCB-DLBCL cell lines with initial low expression of BCL-xL are ideally suited for studying the correlation of high BCL-xL and acquired resistance to chemotherapy [29]. Using a chronic exposure protocol we have described previously [30], we show that SU-DHL-6-199R (S6-R) and OCL-LY-19-199R (OC-R) cells were 4-and 3-fold more resistant, respectively to ABT-199 [31] than parental cells. BCL-xL mRNA levels, as determined by RT-PCR, were more than three-fold higher in ABT-199R OC-R and S6-R as compared to the parental OC and S6 cells (Fig. 1a). As we have previously shown that acquired resistance to ABT-737 occurs via elevated MCL-1 levels that sequester the pro-apoptotic protein BIM [30], leading to a block of apoptosis in response to ABT-737, we examined whether BCL-xL directly mediates ABT-199 resistance by sequestering BIM. Immunoprecipitation of BCL-xL and examination of BIM levels revealed that more BCL-xL-interactive BIM was present in the S6-R cells as compared with the parental cells [31], indicating its functional importance in regulating apoptosis resistance.

### BCL-xL is regulated at the post-transcriptional level by miR-377

To address the molecular mechanism that mediates high BCL-xL RNA levels in resistant cells, we first determined whether BCL-xL is regulated at the transcriptional level by examining activation of the known BCL-xL regulatory transcription factors, STAT3 and NF-κB [32]. As these transcription factors were not activated in our ABT-199R cells (data not shown), it is less likely that the high BCL-xL expression observed is a result of transcriptional regulation.

We next addressed the possibility that altered BCL-xL RNA stability is controlled by a miRNA. Using target prediction software (microRNA.org) to identify miRNAs that have a putative BCL-xL target, we found that miR-377 had the highest score rank of all candidates (Table 1). We decided to focus on miR-377 for two reasons: (i) the prediction analysis identified two complementary sequences in the 3’-UTR of *BCL-xL* mRNA that miR-377 is likely to base-pair with (Additional file 1: Figure S1A), thus suggesting that it is a potential target, and (ii) miR-377 is located at 14q32, the deleted chromosome 14 region that has been described in B-cell lymphomas [33], suggesting that miR-377 may function as a tumor suppresser gene. To test whether miR-377 mediates BCL-xL expression, we first examined whether its expression was associated with that of miR-377. Indeed, expression of miR-377 inversely correlated with that of *BCL-xL* in ABT-199R cells (Fig. 1b).

**Table 1:** miRNAs that target BCL-xL as ordered by sum of mirSVR scores (microRNA.org)

Rank	miRNA
1	miR-377
2	miR-342
3	miR-491
4	let-7i
5	let-7a

### BCL-xL is a direct target of miR-377

Bioinformatics analysis of the *BCL-xL* 3’-UTR using RNAhybrid and miRbase predicted two potential binding sites for miR-377 at positions 1238 and 1412 (Additional file 1: Figure S1A). To examine whether BCL-xL is a direct target of miR-377, we monitored its expression using a 3’-UTR luciferase reporter assay to examine whether the observed reduction in BCL-xL expression during miR-377 up-regulation is a result of a direct targeting of its 3’-UTR by miR-377. We thus cloned a region of *BCL-xL* 3’-UTR (1107 to 1491 nucleotides) containing both of the predicted binding sites downstream of the stop codon of the firefly luciferase open reading frame (Fig. 1c). We also generated mutants (MUT) of miR-377 target sites (Fig. 1d). In the C1 and C2 3’-UTR mutants, seven nucleotides 1238–1244 and 1412–1418, respectively of the target site were mutated to disrupt miR-377 interaction in the predicted seed region (Fig. 1e). In the third C3 mutant (double 3’-UTR mutant), we combined both upstream and downstream miR-377 seed region mutations in order to simultaneously disrupt the miR-377/BCLxL interaction at both sites (Fig. 1e). Each of these constructs was co-transfected with either a miR-377 or a negative control mimic in CHO-K1 cells, with renilla luciferase used as an internal control, and the luciferase activity was measured after 48 h. Ectopic miR-377 mimic expression down-regulated the wild-type (WT) 3’-UTR-associated luciferase activity by ~ 46 % as compared with the negative control mimic (Fig. 1f). Cells transfected with C1- and C2 3’-UTR luciferase reporter and the miR-377 mimic showed reversal of this repression in reporter activity by 22 and 36 %, respectively (Fig. 1f). Remarkably, in cells transfected with the C3 double mutant 3’-UTR luciferase reporter, miR-377 mimic expression was unable to suppress luciferase activity at all. These cells exhibited a complete reversal of luciferase activity, which indicates a direct binding of miR-377 to the predicted target sites in the *BCL-xL* 3’-UTR (Fig. 1f). Taken together, these data demonstrate that the predicted target sites in the *BCL-xL* 3’-UTR are authentic and specific binding sites for miR-377 and, therefore, provide support to our discovery of *BCL-xL* as a direct target of miR-377.

Using both mFold and Nupack RNA folding algorithms, we analyzed the contextual/local secondary structures around the predicted miRNA target site in the short and full length 3’-UTR of *BCL-xL* transcript to eliminate the possibility that different secondary structures may influence miRNA binding. Thus, the contextual structure between the secondary structure of the short and the full-length *BCL-xL* 3’-UTRs, as predicted by mFold RNA folding algorithm, appears to be very similar as both contain six varied size bulges and an almost similar number of helices (Additional file 1: Figure S1B). Therefore, irrespective of the size of the 3’-UTR fragment utilized for target validation assay, our analyses support our above findings that *BCL-xL* is a target of miR-377. As a second independent approach to experimentally confirm that miR-377 is involved in the regulation of *BCL-xL*, a gain-of-function experiment indicated that adding miR-377 mimics at a concentration as low as 50 nM led to a substantial decrease in endogenous BCL-xL protein expression, thus confirming that miR-377 is a direct target of BCL-xL (Fig. 1g).

### miR-377-dependent BCL-xL regulation drives resistance to ABT-199

**Fig. 2:**
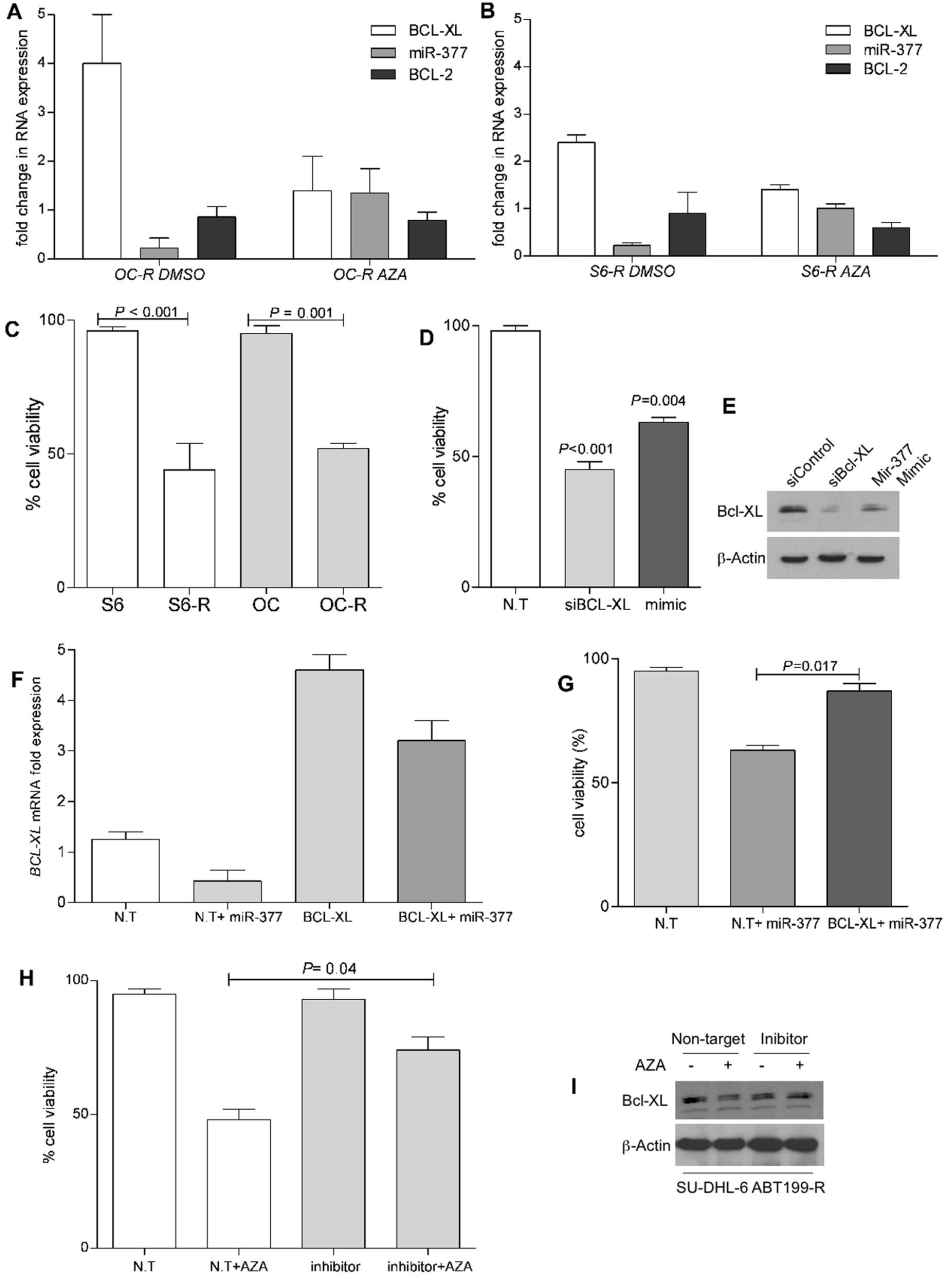
BCL-xL and miR-377 co-regulation mediates ABT-199 resistance. RT-PCR analysis of *BCL-xL*, miR-377, and BCL-2 expression levels after treatment with 5-Aza for 72 h in (**a**) OC-R and (**b**) S6-R cells. *BCL-xL, miR-377,* and *BCL-2* expression levels in OC-R and S6-R were normalized to those in parental OC and S6 cells. DMSO treatment and BCL-2 expression served as negative controls. (**c**) Parental OC, S6 and ABT-199R OC-R and S6-R cells were treated with 5-Aza and cell viability was assessed after 72 h by annexin-V/FITC and propidium iodide staining and analyzed by flow cytometry. (**d**) Cell viability, analyzed by flow cytometry and (**e**) BCL-xL expression, assessed by immunoblot after transfecting S6-R cells with 200 nM of Non-Target (N.T), miR-377 mimic, or siBCL-xL. S6-R cells were transfected with 1 μg of pMIG-Bcl-xL expression plasmid and 24 h later transfected again with 200 nM of miR-377 mimic. After an additional 24 h, analysis of BCL-xL expression by RT-PCR (**f**) and cell viability (**g**) were determined. BCL-xL expression and cell viability were normalized to untransfected S6-R cells. Cell viability (**h**) and BCL-xL expression (**i**) were assessed in S6-R cells treated with 5-Aza for 24 h and then at 48 h following transfection with miR-377 inhibitor (72 h total time). Significance was determined by a *t*-test

To address the molecular mechanism that mediates down regulation of miR-377 in ABT-199- resistant cell lines, we hypothesized that miR-377 might be epigenetically silenced, as several studies reported that miRs are regulated by methylation [34–36]. Indeed, treatment of ABT-199R cells with the DNA-hypomethylating agent 5-Aza-2′-deoxycytidine (5-Aza) for 72 h led to re-expression of miR-377, which was also associated with a decrease in BCL-xL expression (Fig. 2a and b), indicating a tight regulation at the molecular level between BCL-xL and miR-377 expression. Interestingly, ABT-199R cells were highly sensitive to 5-Aza as compared to parental cells (Fig. 2c), indicating that targeting BCL-xL by re-expression of miR-377 can significantly increase cell death (*P* < 0.005) of ABT-199R cells. Moreover, targeting BCL-xL in S6-R cells by transfecting either miR-377 mimics or siBCL-xL led to decrease in BCL-xL expression and significant increase (*P* < 0.005) in cell death (Fig. 2d and e). Importantly, expression of BCL-xL significantly increased cell viability (*P* = 0.017) in miR-377 mimic-treated S6-R cells (Fig. 2f and g). These results indicate that BCL-xL expression can rescue cell viability after treatment with the miR-377 mimic.

To demonstrate that re-expression of miR-377 by 5-Aza impacts on BCL-xL expression, we sought to inhibit miR-377 function with a miR-377 inhibitor consisting in a single-strand reverse complement to the mature miR-377 strand, which is used for preventing its binding to endogenous targets. We therefore treated S6-R cells with 5-Aza for 24 h and then transfected them with the miR-377 inhibitor and then assessed cell viability and BCL-xL expression after 48 h (72 h total time of treatment). Our data indicate that targeting miR-377 led to a substantial blunting of the effect of 5-Aza, as indicated by a significant decrease in cell death (*P* = 0.04) and BCL-xL expression (Fig. 2h and i).

### High BCL-xL expression correlates with low miR-377 expression in B-cell lymphoid malignancies, including CLL patients

**Fig. 3:**
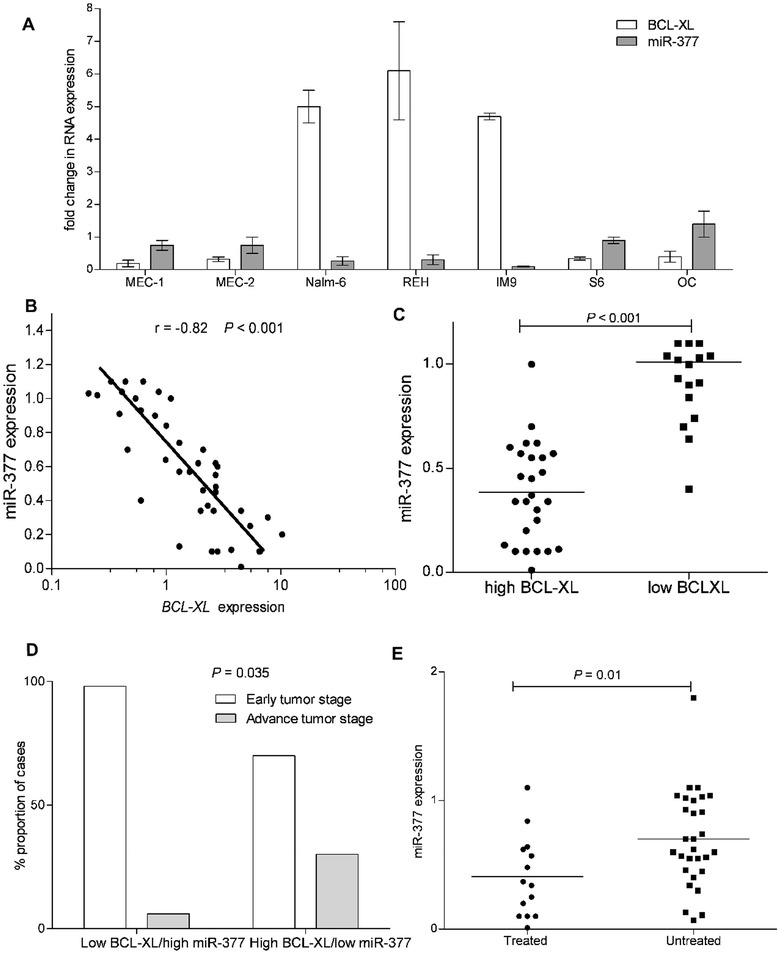
miR-377 expression is inversely correlated with that of *BCL-xL*. **a** Correlation of *BCL-xL* and *miR-377* expression in a panel of lymphoid B-cell lines. *BCL-xL* and miR-377 RNA expression was normalized to that of lymphocytes isolated from healthy donors. **b** miR-377 expression is inversely correlated with that of *BCL-xL* in 43 CLL patients as determined by RT-PCR. Spearman correlation (*r*) and *P* values are shown. **c** CLL patients with high *BCL-xL* levels show decreased levels of miR-377 expression as determined by RT-PCR. Each dot represents one CLL patient. **d** CLL patients with advanced tumor stage have significantly lower miR-377 and higher BCL-xL expression compared to CLL patients with early tumor stage that have high miR-377 and lower BCL-xL expression. **e** miR-377 expression in CLL patients who had received a diverse array of chemotherapy regimens versus untreated CLL patients

We next addressed the generality of the association of BCL-xL and miR-377 expression in a panel of lymphoid B-cell lines. Remarkably, high BCL-xL expression inversely correlated with low miR-377 expression in the seven cell lines examined (Fig. 3a). Importantly, expression of miR-377 inversely correlated (*P* < 0.001, *r* = −0.82) with that of *BCL-xL* in primary CLL patient samples (Fig. 3b and c). Interestingly, we also found that cell lines that have high BCL-xL and low miR-377 expressions are highly resistant to ABT-199 (Additional file 2: Figure S2). In addition, two out of the three cell lines that have high BCL-xL and low miR-377 expression were established from relapsed/higher tumor stage patients.

Next, we wanted to test whether expression of *BCL-xL* and *miR-377* correlate with the severity of the clinical course in CLL patients. Indeed, CLL patients with high *BCL-xL/low miR-377* have more advanced tumor stage than CLL patients with low *BCL-xL/high miR-377* expression (Fig. 3d). Importantly, CLL patients that were previously treated with a wide range of chemotherapeutic regimens had decreased miR-377 expression as compared to untreated CLL patients (Fig. 3e), indicating that exposure to chemotherapy might trigger transcriptional silencing of miR-377, which results in high levels of BCL-xL. These finding may reveal a new mechanism of resistance, in which transcriptional silencing of miR-377, which results in high levels of BCL-xL promotes resistance to chemotherapy.

### miRNAs in the 14q32 cluster region are down-regulated in therapy-resistant cells

**Fig. 4:**
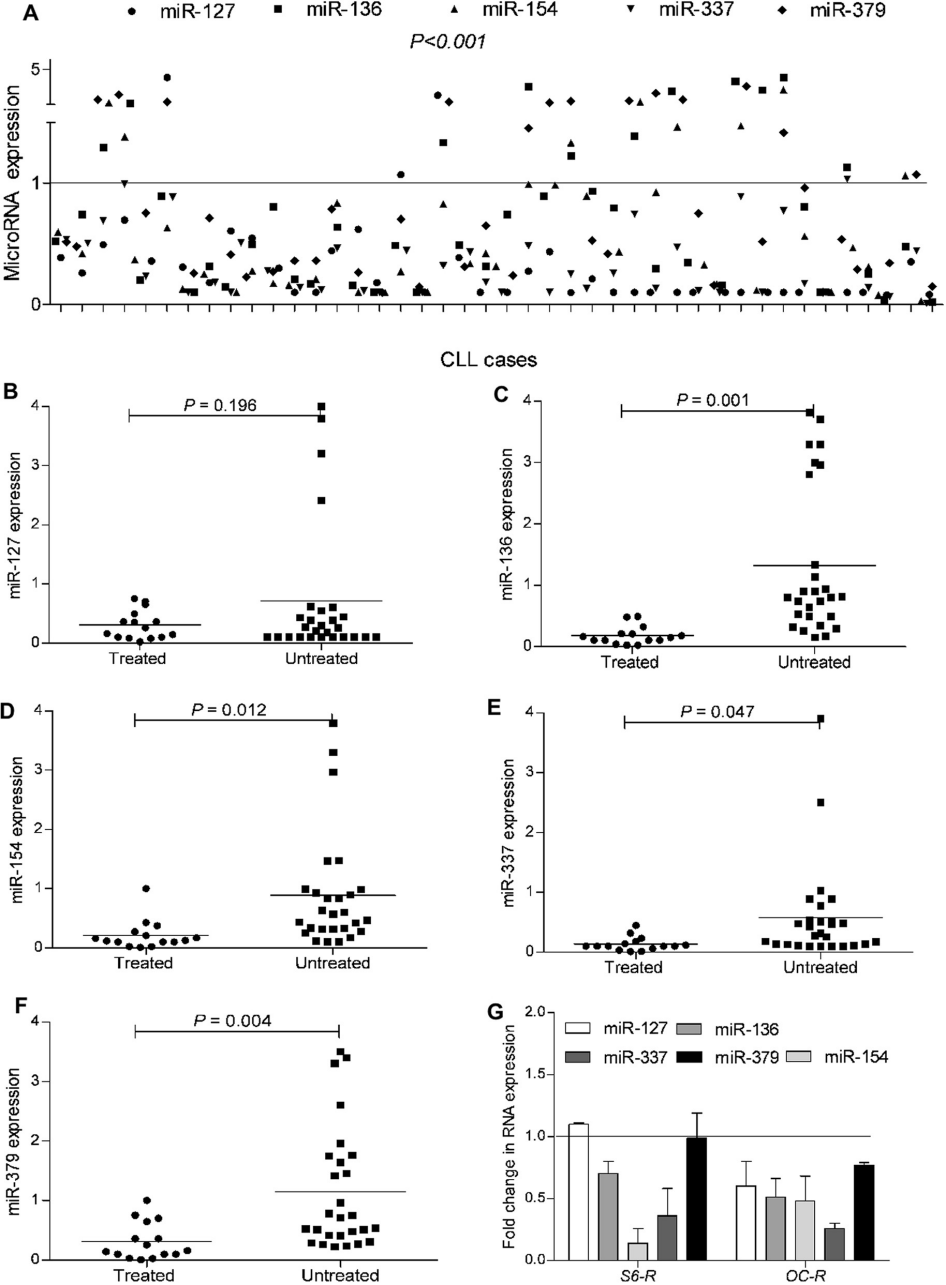
Five miRNAs in the 14q32.31-32 clusters are coordinately down regulated in CLL patients. **a** Expression of five miRNAs, as determined by quantitative RT-PCR in CLL patients. Each dot represents one CLL patient. miRNA expression for each transcript was normalized to that of lymphocytes isolated from healthy donors as indicated by the strait line (corresponding to value 1). Significance was determined by a *t*-test. Expression levels of five miRNAs in treated and untreated CLL patients for **b** miR-127, **c** miR-136, **d** miR-154, **e** miR-337, and **f** miR-379. **g** Expression of five miRNAs in 14q32.31 and 14q32.32 clusters, as determined by quantitative RT-PCR in ABT-199R cell lines. miRNA expression for each transcript was normalized to that obtained in lymphocytes isolated from healthy donors, as indicated by the strait line

It has been previously shown that miR-377 is located at 14q32.31. This region on the long arm of chromosome 14 encodes one of the largest miRNA clusters in humans and is classified into two adjacent miRNA clusters:14q32.31 and 14q32.32 [37]. Therefore, we wanted to test whether the reduced expression of miR-377 is unique or reflects a broader silencing of the mIRs located in these two adjacent clusters. To test this, we randomly selected five additional mIRs located in these two clusters: miR-127, miR-136, miR-154, miR-337, and miR-379 to examine their expression in our CLL patients. Our data indicate that majority of these miRs were significantly down-regulated (*P* < 0.001) in most CLL patients (Fig. 4a). Interestingly, we found that four out these five mIRs had significantly decreased expression in chemothrapy-treated CLL patients as compared to untreated CLL patients (Fig. 4b to f). These findings are consistent with our miR-377 data, which suggest that exposure to chemotherapy might trigger transcriptional silencing of the miRs located in the 14q32 region. Similar differences in the expression of these mIRs were seen in the ABT-199R cell lines compared to parental S6 and OC cells (Fig. 4g).

### Previously treated CLL patients show higher BCL-xL expression

**Fig. 5:**
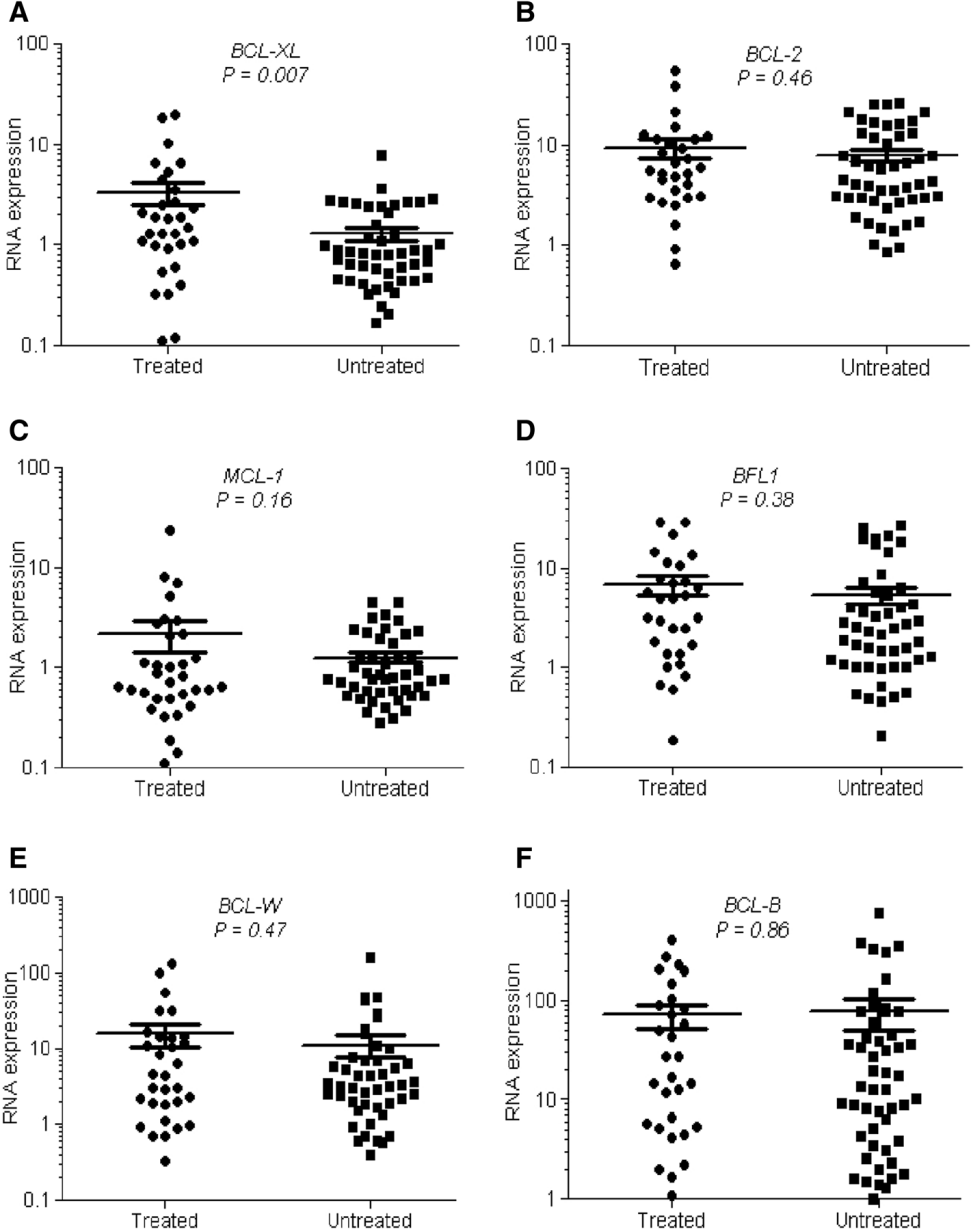
High levels of *BCL-xL* expression in CLL patients previously treated with chemotherapy compared to those untreated. mRNA expression levels in treated and untreated CLL patients for **a** *BCL-xL*, **b** *BCL-2*, **c** *MCL-1*, **d** *BFL-1*, **e** *BCL-W*, and **f** *BCL-B*, determined by RT-PCR. Significance was determined by a *t*-test. RNA expression for each transcript was normalized to that obtained in lymphocytes isolated from healthy donors. Each dot represents one CLL patient

The current standard of care for CLL patients involves sequential cycles of chemotherapy - most often purine analogs or alkylating agents - in combination with an anti-CD20 monoclonal antibody. As almost all CLL patients will eventually relapse, development of acquired resistance to chemotherapy is a significant clinical problem. As 31 of our 76 patients had received a diverse array of chemotherapy regimens, including fludarabine, rituximab, bendamustine, chlorambucil, prednisone, and alemtuzumab, we compared the expression of anti-apoptotic BCL-2 family genes in untreated compared to previously treated patients (Fig. 5). Among all the anti-apoptotic *BCL-2* family genes tested, only *BCL-xL* expression was significantly higher (*P* = 0.007) in CLL patients who received chemotherapy as compared to those who were never treated (Fig. 5a). This result indicates that regardless of the type of therapy used, BCL-xL might confer resistance to conventional cytotoxic chemotherapy.

### High BCL-xL expression is associated with shorter treatment-free survival in CLL patients

**Fig. 6:**
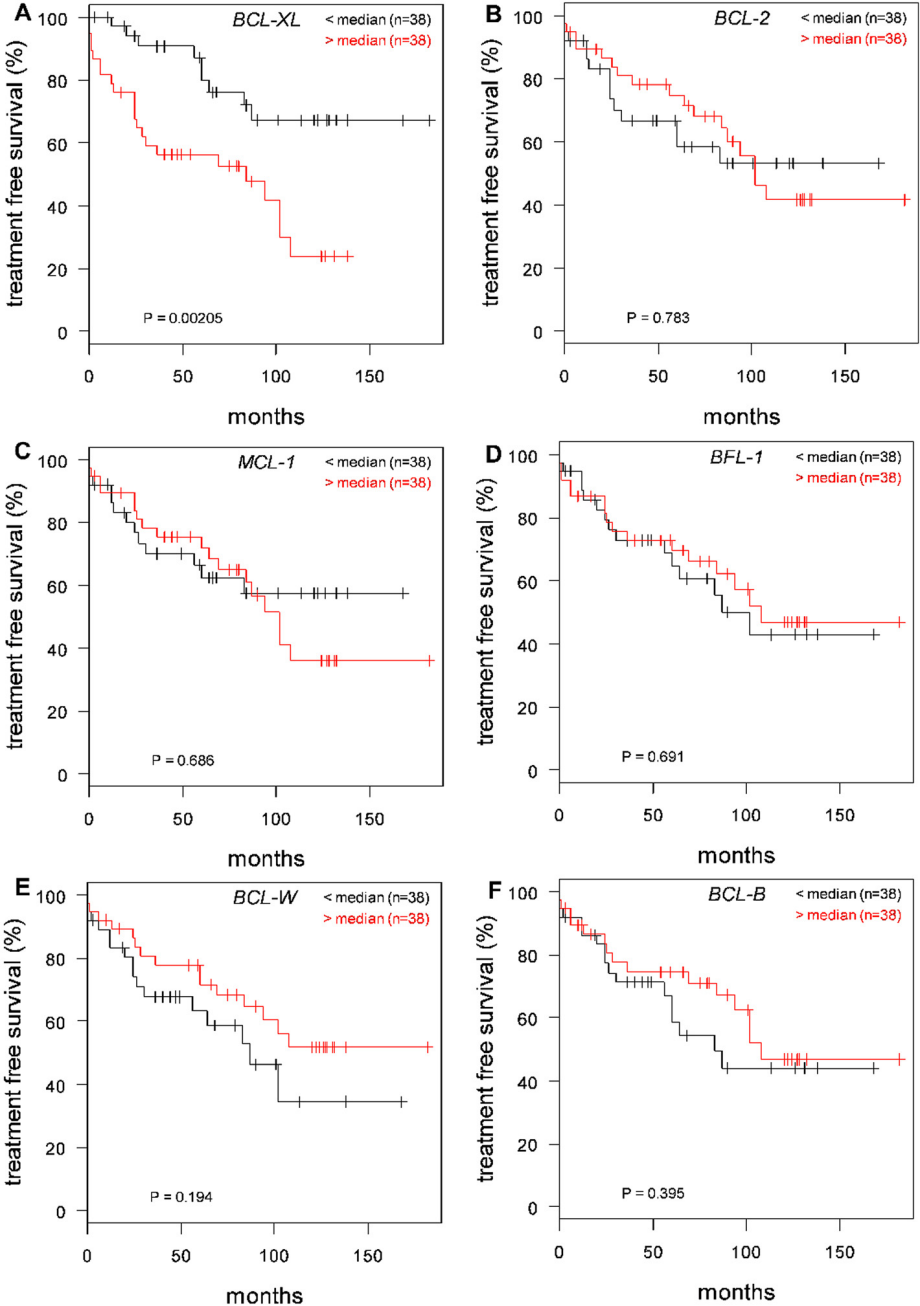
High level of expression of BCL-xL, but not other BCL-2 family members, correlates with treatment-free survival of CLL patients. Kaplan-Meier curves for correlation of treatment-free survival in CLL patients with expression of: **a** *BCL-xL*, **b** *BCL-2*, **c** *MCL-1*, **d** *BFL-1*, **e** *BCL-W*, and **f** *BCL-B* mRNA levels were determined by RT-PCR. *P* values shown are for the log-rank test. RNA expression for each transcript was normalized to that obtained in lymphocytes isolated from healthy donors

**Fig. 7:**
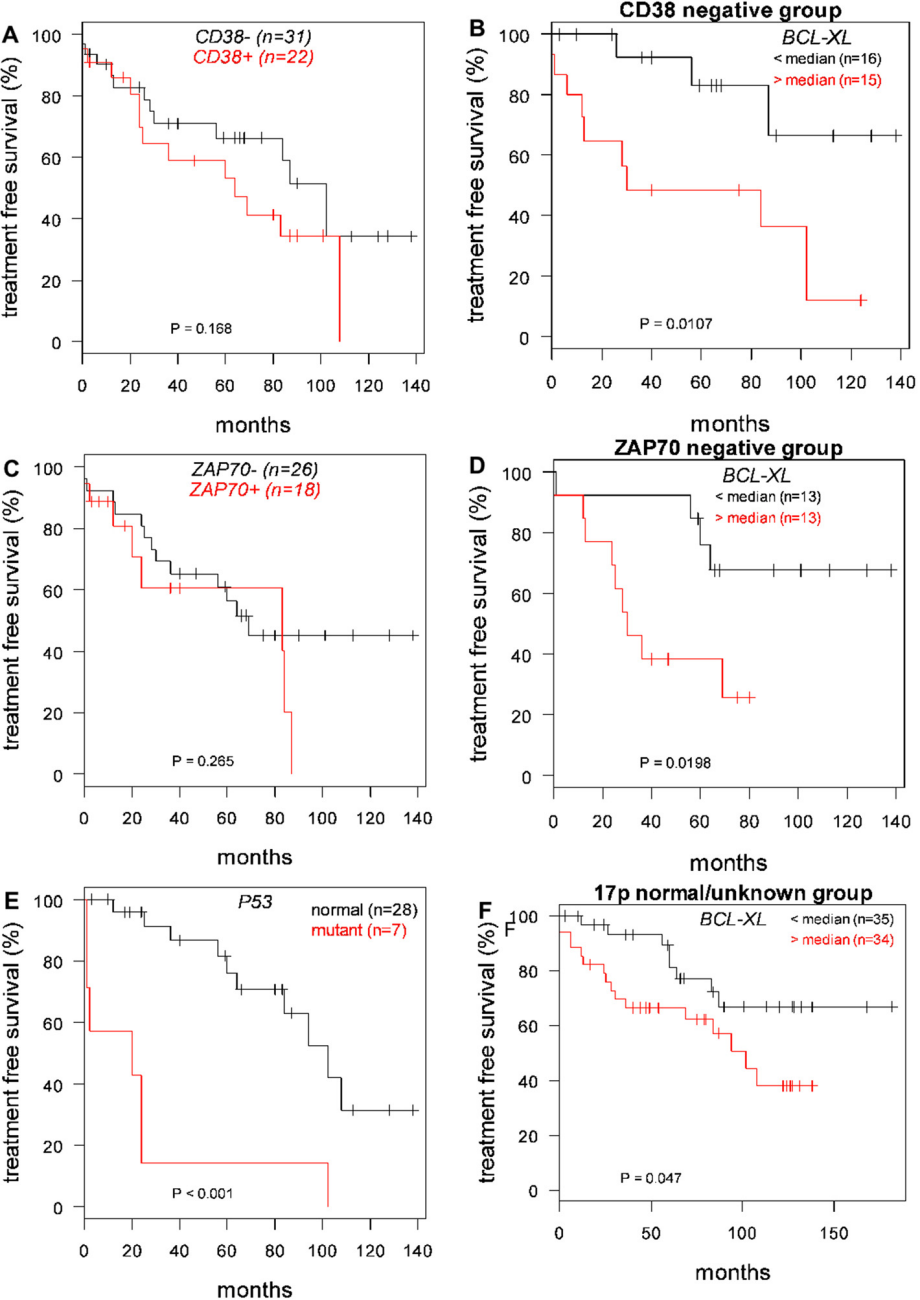
*BCL-xL* expression identifies CLL patients at high risk for short time to treatment who have negative-prognostic markers. Kaplan-Meier curves for correlation of treatment-free survival in CLL patients with various prognostic markers: **a** CD38 positive or negative, **b** CD38 negative with high *BCL-xL* expression, **c** ZAP70 positive or negative, **d** ZAP70 negative with high *BCL-xL* expression, **e** 17p deletion or 17p normal/unknown group **f** 17p normal/unknown group with high *BCL-xL* expression. Log-rank test *P* values are shown

Baseline characteristics of the 76 patients with CLL studied are summarized in Table 2. To further investigate whether expression of anti-apoptotic genes, including BCL-xL, contribute to treatment free survival in CLL patients, RT-PCR was used to assess expression of the anti-apoptotic, *BCL-2*, *MCL-1*, *BCL-xL*, *BFL-1*, *BCL-W*, and *BCL-B* and pro-apoptotic, *BIM*, *PUMA*, and *NOXA* genes. Correlations of the expression of each of these genes with time from diagnosis to start of treatment, i.e., treatment-free survival (Fig. 6, Additional file 3: Figure S3) were examined for significance. We also examined the combined expression of all anti-apoptotic and pro-apoptotic BCL-2 family members and ratios of these (data not shown). Only *BCL-xL* expression emerged as being significantly correlated with treatment-free survival (*P* = 0.002, Fig. 6a). We next assessed the correlation of common CLL prognostic markers p53, ZAP70, and CD38 with treatment-free survival. Patients with p53 (17p) deletion showed very strong correlation with treatment-free survival, whereas ZAP70 and CD38 failed to indicate such correlation (Fig. 7a, c, and e). In contrast, Jonckheere-Terpstra testing showed no significant correlation trend between *BCL-xL* expression and increasing Rai stage, ZAP70-positive/negative, CD38-positive/negative, p53 deletion (17p)/normal status (Additional file 4: Figure S4), or commonly detected chromosomal abnormalities (Table 2), thus indicating that *BCL-xL* expression is independent of known CLL prognostic markers.

**Table 2:** Baseline characteristics of CLL patients

	All Patients		*BCL-XL* < median		*BCL-XL* > median		*P*
			(*n* = 38)		(*n* = 38)		
	Number	Value (range)	Number	Value (range)	No. (%)	Value (range)	
Age, median (range), years		64 (38–90)		64.5 (38–90)		64 (48–87)	N.S.
Sex, male/female	52/24		22/16		30/8		0.08
Rai Stage	*N* = 76						
Early (Stage 0/I/II)	59		36		23		
Advanced (Stage III/IV)	17		2		15		
Zap70 positive	18 of 44		11 of 24		7 of 20		0.937
CD38 positive	22 of 53		10 of 27		12 of 26		0.110
FISH							
17p	7 of 35		1 of 19		6 of 16		0.275
11q	6 of 35		4 of 19		2 of 16		0.666
13q14	22 of 35		12 of 18		10 of 17		0.733
Trisomy 12	6 of 35		2 of 17		4 of 16		0.398

### BCL-xL expression identifies CLL patients at high risk for shorter treatment-free survival time within the negative-prognostic marker groups

Some patients in the ZAP70-negative, CD38-negative, and normal/unknown p53 (17p) status categories also experienced rapid progression and aggressive disease (Fig. 7a, c, and e). *BCL-xL* expression was able to identify patients with high risk within these favorable CLL categories. Remarkably, *BCL-xL* levels could discriminate between patients at high risk who had CD38-negative (*P* = 0.010), ZAP70-negative (*P* = 0.019), or normal/unknown p53 (17p) status (*P* = 0.047), suggesting its potential clinical value (Fig. 7b, d, and f).

## Discussion

CLL undergoes transformation (known as the Richter syndrome), to more aggressive lymphoma, most commonly DLBCL [38]. In our GCB-DLBCL chemotherapeutic resistance model, chronic exposure to ABT-199 up-regulates BCL-xL expression, which is responsible for mediating chemotherapeutic resistance to ABT-199 [31]. In search of the molecular mechanism that mediates high BCL-xL expression, we identified miR-377, expression of which was inversely correlated with that of BCL-xL. In our ABT-199 resistance model, we found that the high BCL-xL expression was due to down-regulation of miR-377, an observation similar to what we found in our CLL patients and a panel of lymphoid B-cell lines. We demonstrate that *BCL-xL* is a direct target of miR-377 by three independent approaches: (i) a luciferase reporter assay, (ii) miR-377 expression modulation both by a mimic and an inhibitor and (iii) over expression of BCL-xL. While little is known about the molecular function of miR-377, in a comprehensive study using integrative genomic approaches, miR-377, among other miRNAs, was found to correlate with advanced tumor stage in solid tumors [39, 40]. Here, we provide evidence for a tight regulation between miR-377 expression and up-regulation of BCL-xL. Based on these data, we propose a novel mechanism by which lymphoid malignant cells regulate BCL-xL and miR-377 expression in order to promote acquired resistance to chemotherapy. Interestingly, BCL-xL is one of the most frequently amplified oncogenes found in solid tumors [41]. Here we identify a novel, miR-377-dependent regulation as an alternative mechanism for BCL-xL expression in leukemic cells.

miR-377 is located on chromosome 14q, with deletion of this site indicating its potential role as a tumor suppressor. 14q deletions are also associated with trisomy 12, a hallmark of CLL [42]. As the frequency of the 14q deletion is relatively low in hematopoietic malignancies, including CLL [33], it is not likely to provide a common therapeutic resistance mechanism. Interestingly, there is a growing interest in the 14q32 chromosomal region because ~ 53 miRNAs are embedded in two adjacent clusters (14q32.31 and 14q32.32); those span more than 200 kb, which were found to be deregulated in various human diseases, including various cancers, both hematologic, such as acute promyelocytic leukemia [43] and of epithelial origin, such as melanoma [44]. These reports support our findings that demethylating agents can restore expression of these miRNAs, including miR-377.

Our ABT-199R model suggests that an alternative, transcriptional silencing mechanism is responsible for the low levels of miR-377 that develop after chronic exposure to ABT-199. Our preliminary experiments using bisulfate genomic sequencing and methylation-specific PCR could not identify differentially methylated regions. Future experiments are needed to delineate how methylation regulates expression of miR-377 and other miRNAs in the 14q32 cluster. Nevertheless, the epigenetic modification of miRNA expression by methylation is well documented. Many miRNAs, including miR-377, are up-regulated following treatment with 5-Aza, a hypomethylating agent approved for use in myeloid, but not lymphoid malignancies [36, 39, 45]. Treatment with 5-Aza led to re-expression of miR-377 leading to decreased BCL-xL expression. Interestingly, unlike parental cells, we found that ABT-199R cells were highly sensitive to 5-Aza. By modulating miR-377 expression with both a mimic and an inhibitor, along with re-expression of BCL-xL, we show that targeting BCL-xL by 5-Aza is a promising approach to overcome ABT-199 resistance. As several hypomethylating agents are currently in clinical use [45, 46], this possibility could be quickly tested in clinical trials.

We show that CLL patients previously treated with a variety of chemotherapeutic regimens had higher BCL-xL and lower miR-377 expression as compared to untreated patients. Moreover, CLL patients with high BCL-xL/low miR-377 expression also have a more advanced tumor stage. These clinically relevant data along with our ABT-199R model suggest that BCL-xL might be critical for conferring general chemotherapy resistance. In support of our findings, other *in vitro* studies also support a role for BCL-xL in promoting chemotherapy resistance. Thus, chemotherapeutic resistance to a group of compounds that repress MCL-1 expression has been linked to high *BCL-xL* mRNA expression [47]. This finding indicates that a patient-selection strategy for development of any MCL-1 inhibitor should focus on patients with low BCL-xL expression. Moreover, resistance to 122 standard chemotherapy agents correlated with high BCL-xL expression in the NCI 60 cell line panel [48].

Although the role of BCL-2 and MCL-1 in the molecular pathogenesis of CLL has been extensively studied, less is known about the role of the other anti-apoptotic BCL-2 family members. Here, we have applied a highly sensitive and quantitative assay to examine the clinical value of expression for all anti-apoptotic BCL-2 family genes. Out of all these gene expression analyses, for individual or different combinations of these genes, only *BCL-xL* expression had clinically relevant predictive value. In our patient cohort, *BCL-2* and *MCL-1* levels did not discriminate between those at risk for short treatment free survival and those who were not, consistent with previous reports [8, 49].

In contrast to p53 (17p) deletions, ZAP70 and CD38 did not show significant correlations with treatment free survival in our patient population. Reports conflict on the significance of ZAP70 and CD38 in predicting clinical outcome in CLL, likely because of the lack of a standardized method for determining what constitutes positive and negative test results [21, 22, 50]. Interestingly, we found that *BCL-xL* expression levels could identify patients at high risk who were negative for ZAP70 or CD38, suggesting the possibility that it is a more robust and/or independent indicator of clinical outcome. While p53 deletions/mutations generally indicate aggressive disease with poor prognosis, the majority of CLL patients, which do not have this abnormality, remain heterogeneous. *BCL-xL* expression levels were able to further stratify this group in terms of time to requiring treatment, indicating that *BCL-xL* may also be useful in this context. Our findings that BCL-xL has prognostic value are not limited to CLL. Similarly, it was reported that high BCL-xL expression correlated with short overall survival in follicular lymphoma [51]. As a further support for the role of BCL-xL in a wide range of B lymphoid malignancies, the clinical outcome of GCB-DLBCL patients is better than of those with non-germinal center-DLBCL, likely also due to the fact that GCB-DLBCL cells have significantly lower BCL-xL expression [29, 52].

## Conclusion

Our study provides new insights into the role of BCL-xL/miR-377 regulation in chemotherapy resistance in B-cell lymphoid malignancies. As anti-apoptotic BCL-2 family protein inhibitors have shown promising results in clinical trials, our findings underscore the importance of determining expression of the anti-apoptotic BCL-2 family genes, not only for choosing the appropriate targeted therapy and chemotherapy combination for each patient, but also as a means of monitoring the expression of these genes during post-chemotherapy follow-up visits.

## Methods

### Cell lines and reagents

Human lymphoid cell lines SU-DHL-6, OCL-LY-19, Nalm-6, Reh, and IM-9 were obtained from ATCC; Mec-1 and Mec-2 were a gift from Dr Yogen Saunthararajah (Cleveland Clinic). All cell lines were cultured in RPMI-1640 medium supplemented with 10 % fetal bovine serum (Atlanta Biologicals, Lawrenceville, GA), and antibiotic-antimycotic (Gibco, Life Technologies, Gaithersburg, MD). Cell lines were routinely screened for mycoplasma, variations in growth rates, changes in morphological characteristics, and their response to stress with Annexin V FITC-PI staining; their passage number did not exceed 20. The development of DLBCL cell lines with acquired resistance to ABT-199 (ABT-199R) was previously described [31]. The ABT199-R cells were routinely tested for resistance to ABT-199 and cultured without drug for 72 h before they were used in experiments.

### Luciferase assays

The wild-type (WT)-3’-UTR reporter plasmid was constructed by cloning a 384-base pair fragment of the *BCL-xL* 3’-UTR spanning the predicted target site for miR-377 downstream of the firefly luciferase coding region in the pMIR-REPORT vector (Ambion, Austin, TX). Site-directed mutagenesis of the putative target sites for miR-377 in the WT-3’-UTR construct was carried out to generate the mutant MUT-3’-UTR constructs. Nucleotide sequences of the constructs were confirmed by DNA sequencing. Luciferase assays were performed as previously described [53]. CHO-K1 (30,000 cells/well) placed in 24-well plates, 1 day later were co-transfected using Lipofectamine 2000 (Invitrogen), with 100 ng WT-3’-UTR or MUT-3’-UTR firefly luciferase reporter constructs, 0.5 ng renilla luciferase reporter plasmid (Promega, Madison, WI) and either miR-377 or negative control (NC) mimics (10 nM). Cell lysates were assayed for firefly and renilla luciferase activities 48 h after transfection using the dual-luciferase reporter assay system (Promega) and a Victor^3^ multilabel plate reader (PerkinElmer). Renilla luciferase activity served as a control for transfection efficiency. Data are shown as the ratio of firefly luciferase activity to renilla luciferase activity. All experiments were performed at least three times in triplicate.

### Transfections to modulate miR-377 and BCL-xL function

The miR-377 mimic sequence 5’-AUCACACAAAGGCAACUUUUGU-3’ and a Non-Target (negative control) were purchased from Qiagen. They were transfected using HiPerFect transfection reagent (Qiagen, Germantown, MD) in 293 T cells according to manufacturer’s protocol. The effect of the miR-377 mimic on BCL-xL levels was validated at the protein level by western blot using an antibody against BCL-xL (Santa Cruz Biotechnology). 200 nM of miR-377 mimic (Qiagen), miR-377 inhibitor (Sigma), pMIG-Bcl-xL expression plasmid [54] (obtained from Addgene), siBCL-xL, or siControl (Santa Cruz Biotechnology) transfection in ABT-199R SU-DHL-6 cells was achieved using the Amaxa Nucleofector Kit V (Lonza, Walkersville, MD, USA) (program number O-007) according to the manufacturer’s protocol, as described earlier [31]. siBCL-xL and siControl consist of pools of 3–5 target-specific 19–25 nt siRNAs. ABT-199R SU-DHL-6 cells were transfected with 1 μg of pMIG-Bcl-xL, followed 24 h later by transfection with 200 nM of miR-377 mimics. After an additional 24 h, BCL-xL expression levels were determined by quantitative RT-PCR.

### Immunoblotting

The cell pellets were lysed with 1 % NP-40 lysis buffer (20 mmol/L Tris–HCl, pH 7.5; 1 mmol/L EDTA; 150 mmol/L NaCl; 1 % NP-40) phosphatase inhibitors cocktail 2 and 3 (Sigma) and containing protease inhibitors (Roche) for 30 to 45 min at 4 °C. Protein lysates were prepared after calculating protein concentration using the Bradford reagent (Biorad) and 50 μg of protein was resolved on 10 % SDS-PAGE followed by transferring to nitrocellulose (Millipore) [30]. Immunoblotting was performed with primary antibodies against BCL-xL (Santa Cruz Biotechnology) and β-actin (Sigma). The secondary anti-mouse antibodies were purchased from Thermo-Fisher Scientific.

### Purification of primary CLL cells and lymphocytes from healthy donors

Fluorescence in situ hybridization was used as part of the diagnostic evaluation of CLL patients [11]. Peripheral blood samples from 76 patients with CLL and six healthy donors were obtained with the patients’ informed consent according to protocols approved by the Cleveland Clinic Institutional Review Board according to the Declaration of Helsinki. All primary CLL cells were freshly processed without freezing. Lymphocytes were purified by Ficoll-Paque PLUS (GE Healthcare) gradient centrifugation. A lymphocyte sample set isolated from 6 healthy donors was used to establish a baseline comparison between mRNA levels in CLL and healthy donors, with highly purified lymphocytes obtained using the Gambro Elutra Cell Separation System [11].

### Flow cytometry

Cell death was assessed by phosphatidylserine externalization. Cell lines were stained with fluorescein-conjugated annexin V (BD Biosciences) and propidium iodide and analyzed on a BD FACSCalibur flow cytometer. Raw data were analyzed using the CellQuest Version 5.2.1 software. Results were normalized to survival of untreated cells. Flow cytometric immunophenotyping using fluorescently labeled monoclonal antibodies against ZAP70 and CD38 was performed at the Cleveland Clinic as part of diagnostic evaluation on a FACSCanto instrument (BD Biosciences). Staining protocols were standard lyses/washing protocols, as previously described [11].

### RNA isolation and quantitative RT-PCR

Total RNA was isolated using the Trizol method (Invitrogen). 1 μg of RNA samples were reverse transcribed using the TaqMan reverse transcription kit and amplified using the SYBR Green Master Mix (Applied Biosystems) and examined on a 7500 Real-Time PCR system (Applied Biosystems). Quantitative, real-time reverse transcriptase polymerase chain reaction (RT-PCR) with the specific BCL-2 family primers was performed as we have described previously [11], using the respective primers for *BCL-2* family and β-actin as a control. For the 5-aza-2’-deoxycytidine (5-Aza) experiment, RNA was isolated from ABT-199-resistant (ABT-199R) cell lines SU-DHL-6 and OCL-LY-19 treated with 5 μM 5-Aza (Sigma) for 72 h and expression determined by RT-PCR.

### miRNA amplification

Megaplex™ RT Primers (Applied Biosystems), which are 380 stem-looped reverse transcripts that enable the synthesis of cDNA for mature miRNAs, were used. The TaqMan MicroRNA Reverse Transcription Kit (Applied Biosystems) was used to make cDNAs for mature miRNAs. The SYBR Green Master Mix (Applied Biosystems) was used to amplify miR-377, using specific miR-377 primers [55] 5’-GAGCAGAGGTTGCCCTTG-3’ (forward) and 5’-ACAAAAGTTGCCTTTGTGTGA-3’ (reverse). The U6 small nuclear RNA primers[55] 5’-CTCGCTTCGGCAGCACA-3’ (forward) and 5’-AACGCTTCACGAATTTGCGT-3’ (reverse) were used as an internal control.

### Statistical analyses

The Cox proportional hazards model was used to identify significant survival dependencies on covariates. To illustrate associations, Kaplan-Meier curves were plotted using covariates dichotomized at their medians and compared for significant differences using the log rank test. The Jonckheere-Terpstra test for trend was used to determine whether BCL-xL expression was related to increase in Rai stage. Two sample t-tests were used to compare advance tumor stages (stages III/IV) vs. early tumor stage (stages 0/I/II), BCL-xL expression in treated vs. untreated, ZAP70, CD38, and p53 (17p) status in CLL patients. The Spearman correlation was used to assess the strength and direction of association between BCL-xL and miR-377 expression. All computations were performed in R. *P* < 0.05 was used to indicate statistical significance. Patient records were extracted for dates of diagnosis and date of initiation of CLL-specific therapy in order to calculate time to progression to disease requiring treatment.

## Additional files

Additional file 1: Figure S1. BCL-XL mRNA structure. (A) The 3’-UTR mRNA of *BCL-XL* contains two predicted miR-377 binding sites*.* (B) The contextual/local secondary structures around the predicted miRNA target site in the short and full length 3’-UTR of Bcl-xL transcript as predicted by mFold and Nupack RNA folding algorithms. (JPEG 446 kb) Additional file 2: Figure S2. Lymphoid B-cell lines with low miR-377/high BCL-XL expression are more resistant to ABT-199. Cell viability of a panel of lymphoid B-cell lines after treatment with 200 nM of ABT-199. (JPEG 105 kb) Additional file 3: Figure S3. Kaplan-Meier curves for correlation of treatment-free survival with pro-apoptotic *BCL-2* expression levels. (A) *PUMA*, (B) *NOXA*, and (C) *BIM. P* values shown are for the log-rank test. (JPEG 309 kb) Additional file 4: Figure S4. Correlation of BCL-XL expression and CLL prognostic markers. *BCL-XL* expression, as determined by quantitative RT-PCR, was plotted against (A) Rai stage, (B) p53 (17p), (C) ZAP70, and (D) CD38. Significance was determined by a *t*-test. (JPEG 281 kb)

## Abbreviations

3’-UTR: 3’-untranslated region
5-Aza: 5-Aza-2’-deoxycytidine
ABT-199R: ABT-199-resistant cell lines
BH3: BCL-2 homology domain 3
CD-38: cluster of differentiation 38
CLL: chronic lymphocytic leukemia
DLBCL: diffuse large B-cell lymphoma cells
GCB: germinal center B cells
IGHV region: immunoglobulin heavy chain variable region
miR-377: microRNA-377
miRs: microRNAs
MUT: mutants
P53: tumor suppressor protein 53
RT-PCR: reverse transcription polymerase chain reaction
siRNA: small interfering RNA
ZAP-70: zeta-chain-associated protein kinase 70
WT: wild-type
