# Supplementary figures and images for: miR-377-dependent BCL-xL regulation drives chemotherapeutic resistance in B-cell lymphoid malignancies
Source: Mol Cancer. 2015 Nov 4;14:185. doi: 10.1186/s12943-015-0460-8 (PMC4632834; doi:10.1186/s12943-015-0460-8)

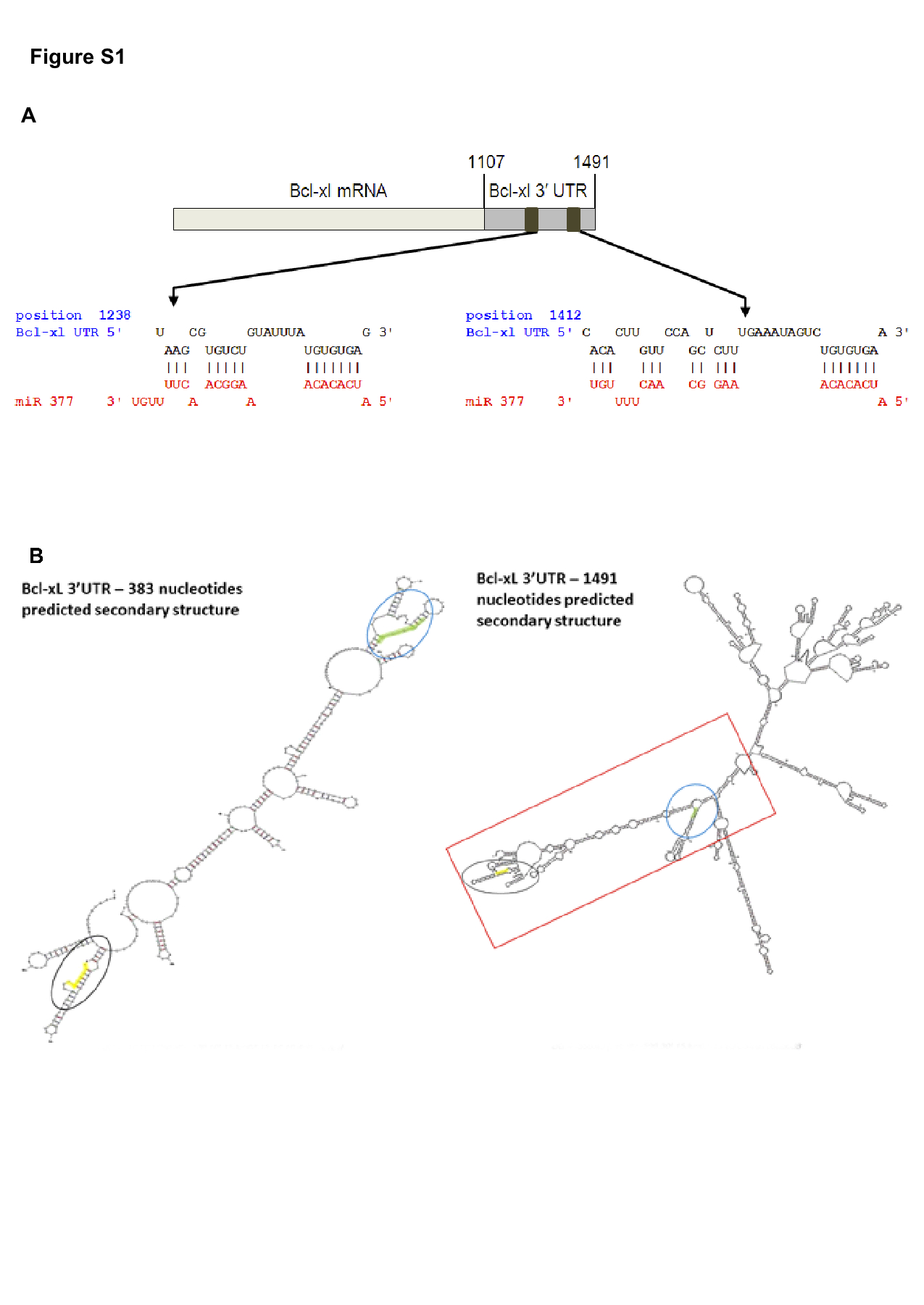

Supplement: Additional file 1: Figure S1. — BCL-XL mRNA structure. (A) The 3’-UTR mRNA of BCL-XL contains two predicted miR-377 binding sites. (B) The contextual/local secondary structures around the predicted miRNA target site in the short and full length 3’-UTR of Bcl-xL transcript as predicted by mFold and Nupack RNA folding algorithms. (JPEG 446 kb) [file 12943_2015_460_MOESM1_ESM.jpg]

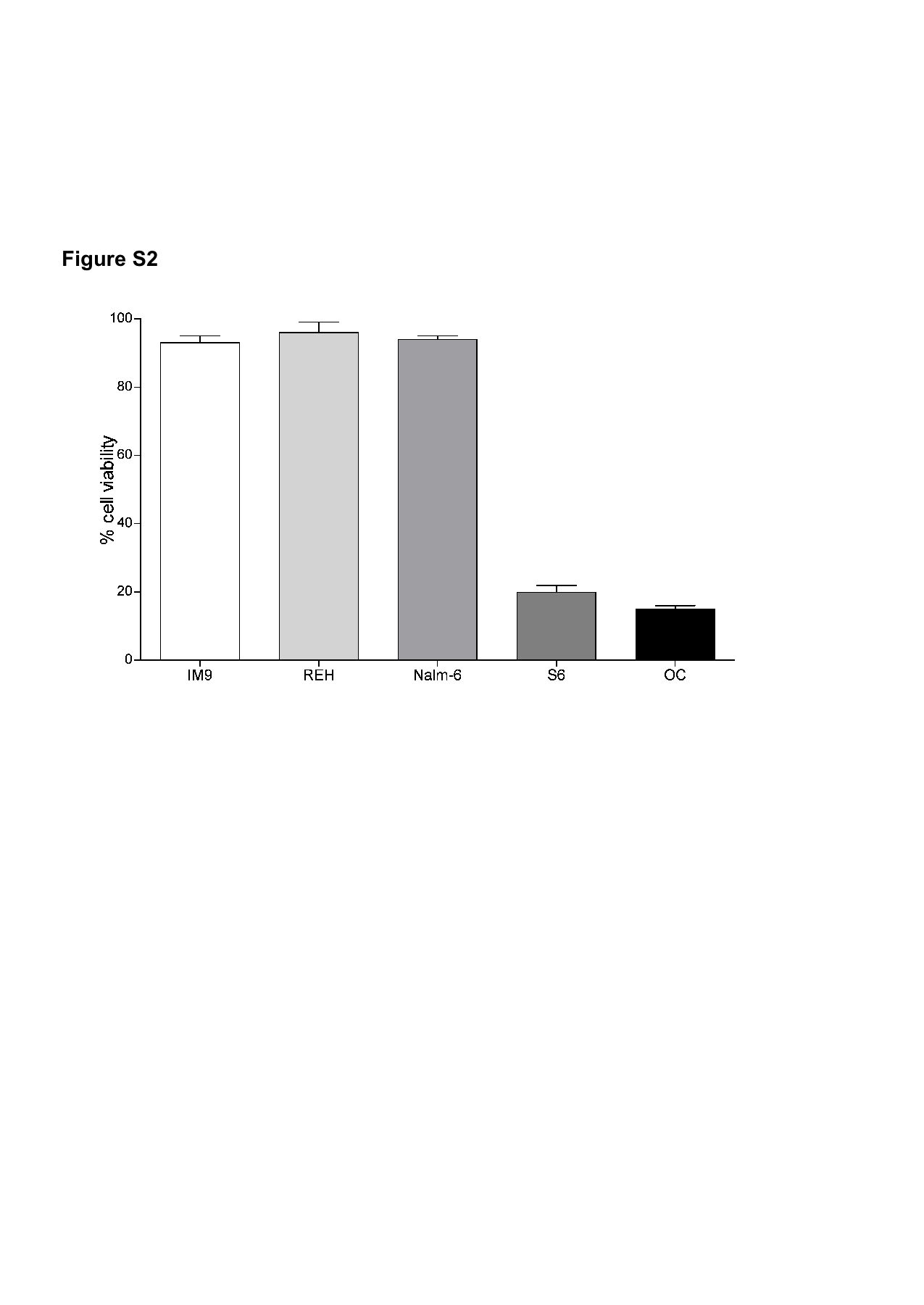

Supplement: Additional file 2: Figure S2. — Lymphoid B-cell lines with low miR-377/high BCL-XL expression are more resistant to ABT-199. Cell viability of a panel of lymphoid B-cell lines after treatment with 200 nM of ABT-199. (JPEG 105 kb) [file 12943_2015_460_MOESM2_ESM.jpg]

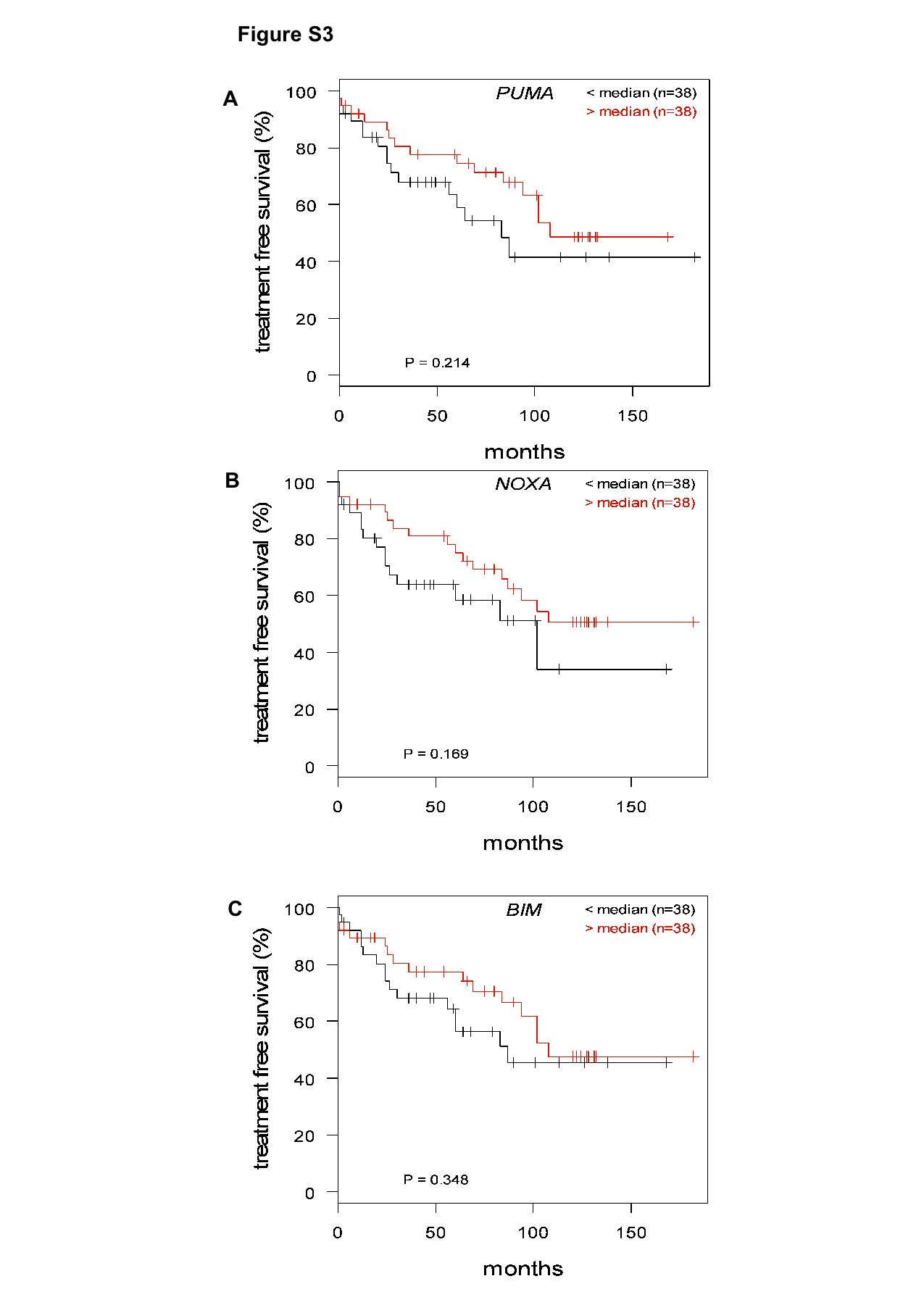

Supplement: Additional file 3: Figure S3. — Kaplan-Meier curves for correlation of treatment-free survival with pro-apoptotic BCL-2 expression levels. (A) PUMA, (B) NOXA, and (C) BIM. P values shown are for the log-rank test. (JPEG 309 kb) [file 12943_2015_460_MOESM3_ESM.jpg]

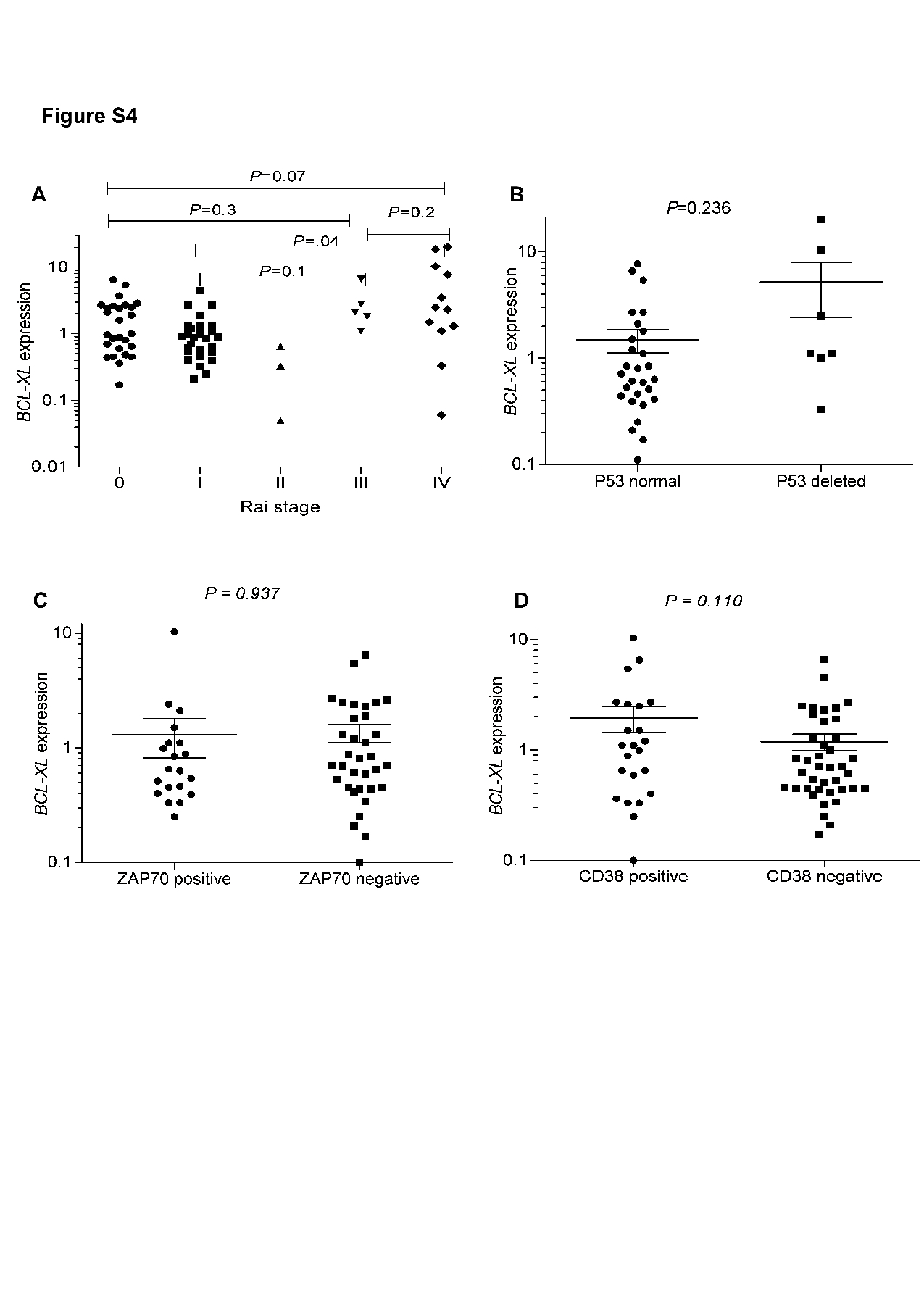

Supplement: Additional file 4: Figure S4. — Correlation of BCL-XL expression and CLL prognostic markers. BCL-XL expression, as determined by quantitative RT-PCR, was plotted against (A) Rai stage, (B) p53 (17p), (C) ZAP70, and (D) CD38. Significance was determined by a t-test. (JPEG 281 kb) [file 12943_2015_460_MOESM4_ESM.jpg]
